# The Contribution of Ionic Currents to Rate-Dependent Action Potential Duration and Pattern of Reentry in a Mathematical Model of Human Atrial Fibrillation

**DOI:** 10.1371/journal.pone.0150779

**Published:** 2016-03-10

**Authors:** Young-Seon Lee, Minki Hwang, Jun-Seop Song, Changyong Li, Boyoung Joung, Eric A. Sobie, Hui-Nam Pak

**Affiliations:** 1 Yonsei University Health System, Seoul, Republic of Korea; 2 Department of Pharmacology and Systems Therapeutics, Icahn School of Medicine at Mount Sinai, New York, New York, United States of America; Indiana University, UNITED STATES

## Abstract

Persistent atrial fibrillation (PeAF) in humans is characterized by shortening of action potential duration (APD) and attenuation of APD rate-adaptation. However, the quantitative influences of particular ionic current alterations on rate-dependent APD changes, and effects on patterns of reentry in atrial tissue, have not been systematically investigated. Using mathematical models of human atrial cells and tissue and performing parameter sensitivity analysis, we evaluated the quantitative contributions to action potential (AP) shortening and APD rate-adaptation of ionic current remodeling seen with PeAF. Ionic remodeling in PeAF was simulated by reducing L-type Ca^2+^ channel current (I_CaL_), increasing inward rectifier K^+^ current (I_K1_) and modulating five other ionic currents. Parameter sensitivity analysis, which quantified how each ionic current influenced APD in control and PeAF conditions, identified interesting results, including a negative effect of Na^+^/Ca^2+^ exchange on APD only in the PeAF condition. At high pacing rate (2 Hz), electrical remodeling in I_K1_ alone accounts for the APD reduction of PeAF, but at slow pacing rate (0.5 Hz) both electrical remodeling in I_CaL_ alone (-70%) and I_K1_ alone (+100%) contribute equally to the APD reduction. Furthermore, AP rate-adaptation was affected by I_Kur_ in control and by I_NaCa_ in the PeAF condition. In a 2D tissue model, a large reduction (-70%) of I_CaL_ becomes a dominant factor leading to a stable spiral wave in PeAF. Our study provides a quantitative and unifying understanding of the roles of ionic current remodeling in determining rate-dependent APD changes at the cellular level and spatial reentry patterns in tissue.

## Introduction

Atrial fibrillation (AF) is a common atrial arrhythmia, especially prevalent among people older than 70 years old, and is the major cause of cardioembolic stroke [1, 2]. When AF occurs in a young person with a structurally normal heart, it tends to appear only intermittently and to terminate spontaneously. Progression of AF, however, causes electrical remodeling of ion channel expression, structural remodeling including fibrosis and gap junctional changes, and autonomic neural remodeling [3–5]. When progression of AF involves electrical ion channel remodeling, the longer AF duration promotes more stable AF maintenance: AF begets AF [3]. Cellular remodeling caused by AF leads to changes in multiple ionic currents and morphological changes in the cellular action potential (AP), including pronounced AP shortening. Persistent AF (PeAF), which is defined as AF lasting longer than 7 days [6], also reduces action potential duration (APD) adaptation, which means that AP shortening caused by rapid pacing is significantly attenuated in patients with longstanding PeAF [3, 7]. Kneller et al. [8] suggested that reduced I_CaL_ can be the underlying factor in the loss of APD rate adaptation in AF-induced electrical remodeling, while Zhang et al. [9] highlighted the effect of increased I_K1_ to shorten APD. For the mechanisms of rate-dependent APD shortening in PeAF, up-regulation of I_K1_ [9–12], reduction in I_CaL_ [7, 13], and changes in intracellular calcium handling [8] have all been suggested in previous studies. Despite extensive previous studies [8, 9, 14], a unifying understanding of quantitative influences of ion currents on rate-dependent changes in APD in human atrium has not been established. Therefore, we quantified the influences of ionic currents on rate-dependent APD and spiral wave reentry in control and AF condition by: (1) computer simulation of a mathematical model of the human atrial cell, (2) a parameter sensitivity analysis of the model, and (3) computer simulation of reentrant waves in two-dimensional atrial tissue under different conditions.

## Methods

### Simulation of a human atrial cell model

We numerically simulated mathematical models of the human atrial cell that was first described by Courtemanche et al. [15] at basic cycle lengths (BCL) ranging from 330 ms to 2000 ms. Stimulation amplitude was set as twice the threshold amplitude for each cycle length. Every simulation with periodic stimulation ran for 250 s. Ionic remodeling in PeAF was taken into consideration by adjusting seven model parameters (Table 1): G_Na_ (−10%), G_to_ (−70%), G_CaL_ (−50% or -70%), G_Kur_ (−50%), [Ca^2+^]_up(max)_ (-20%), G_K1_ (+100%), I_NaCa(max)_ (+40%). We followed the work of Grandi et al. [16] to change model parameters to reproduce PeAF because they adjusted their model parameters through a comprehensive literature review of previous experimental data (Table 1). But it seems that there is no clear consensus about electrical remodeling in I_Na_ in PeAF. Sossalla et al. suggested that peak I_Na_ density decreased by 16% in PeAF [17], whereas Bosch et al. found no change [18].

**Table 1:** Ion current changes in PeAF condition.

Ionic Currents	Model parameter	% change in PeAF from the control	Wilhelms et al. [19]	Pandit et al. [14]
I_Na_	G_Na_	-10% [17]	n/a	n/a
I_to_	G_to_	-70%	-65%	-50%
I_CaL_	G_CaL_	-50% or -70%	-65%	-70%
I_Kur_	G_Kur_	-50%	-49%	-50%
SR leak	[Ca^2+^]_up(max)_	-20%	n/a	n/a
I_K1_	G_K1_	+100%	+110%	+110%
I_NCX_	I_NaCa(max)_	+40%	n/a	n/a

### Multivariable regression method

Parameter sensitivity analysis was performed by randomly varying 20 parameters from the base line values in the model, and running the model for 1000 trials [20–22]. A complete list of 20 parameters varied for the parameter sensitivity analysis and their control values are provided in Table 2. Furthermore, we used a statistical linear regression method to correlate the changes in parameter set (input) to changes in APD which was measured at a fixed threshold value of V = −70.8 mV to find APD_90_ at 1 Hz pacing rate in control. For each trial, we randomly varied model parameters by multiplying the baseline value of each parameter by a log-normally distributed random scale factor. The scale factors had a median value of 1, and the log-transformed scale factors had a standard deviation of 0.1. Regression analysis was performed to calculate sensitivity coefficients between input parameters and output. Randomly varying parameters were placed in an input matrix **X** with dimensions 1000 (trials) by 20 (parameters). We calculated simulated APDs in each of the model variants and placed these values in an output matrix **Y** with dimensions 1000 by 1. The correlation between X and Y was calculated by a linear regression method to obtain a B matrix of the dimensions 20 by 1 such that X*B≈**Y**. To perform all simulations in this study, we used MATLAB (The MathWorks, Natick, MA).

**Table 2:** Courtemanche et al. model parameters used for the sensitivity analysis.

Parameter	Definition	Default Values
G_Na_	Maximal I_Na_ conductance	7.8 nS/pF
G_b,Na_	Maximal I_b,Na_ conductance	0.00113 nS/pF
G_CaL_	Maximal I_CaL_ conductance	0.1238 nS/pF
G_b,Ca_	Maximal I_b,Ca_ conductance	0.00113 nS/pF
G_to_	Maximal I_to_ conductance	0.1652 nS/pF
G_K1_	Maximal I_K1_ conductance	0.09 nS/pF
G_Kr_	Maximal I_Kr_ conductance	0.0294 nS/pF
G_Ks_	Maximal I_Ks_ conductance	0.129 nS/pF
G_Kur_	Scale factor of I_Kur_ maximal conductance	1
I_NaK(max)_	Maximal I_NaK_	0.60 pA/pF
I_NaCa(max)_	Maximal I_NaCa_	1600 pA/pF
K_m,Ca_	[Na^+^]_o_ half-saturation constant for I_NaCa_	1.38
K_m,Na_	[Ca^+^]_o_ half-saturation constant for I_NaCa_	87.5 mM
γ	Voltage dependence parameter for I_NaCa_	0.35
K_sat_	Saturation factor for I_NaCa_	0.1
[Ca^2+^]_up(max)_	Maximal Ca^2+^ concentration in uptake compartment	15.0 mM
I_up(max)_	Maximal I_up_	0.005 mM/ms
k_rel_	Maximal release for I_rel_	30.0 ms^-1^
K_up_	[Ca^2+^]_i_ half-saturation constant for I_up_	0.00092 mM
I_p,Ca(max)_	Maximal I_p,Ca_ (sarcolemmal Ca^2+^ pump)	0.275 pA/pF

### Calculation of rate-dependent electric charge difference during AP

Quantitative contributions of individual ionic currents to AP rate-dependence was calculated by the method of Cummins et al. [23]. Because a single AP involves multiple ionic currents of either inward or outward directions, the integration of a single current (Q) will be the total charge carried by a current during the AP. Thus, inward currents will have a positive Q and outward currents will have a negative value. We define ΔQ as the difference of total charge from 2 Hz to 0.5 Hz pacing rates.

$$Q^{i}=\int_{tstim}^{trepol}I_{ion}^{i}dt$$

$$\Delta Q^{i}=Q^{i}{}_{0.5Hz}-Q^{i}{}_{2Hz}$$

where i denotes channel type. We set t_stim_ for the time of stimulus applied at t = 20 ms and t_repol_ measured at rest (t = 500 ms). Thus, currents with negative (positive) ΔQ contribute to APD prolongation (shortening), respectively.

### Rate-dependent APD

Rate-dependent APD adaptation was represented by displaying the curves of steady-state APD vs BCL by stimulating the cell model with pacing rates from 0.5 Hz to 3 Hz. The curves show the steady-state APD as a function of pacing rate. We chose four different cases: the control case, I_CaL_ with 50% down-regulation [24], I_K1_ with 100% up-regulation [10], and the AF condition as given in Table 1. In particular, we considered three individual cases, I_CaL_ with 50% down-regulation, 70% down-regulation, and I_K1_ with 100% up-regulation, as these were suggested as having important roles in the reduction of APD [7, 11, 12, 25].

### Simulation of 2D tissue model

We modeled electrical activity of the atrial cells in two-dimensional isotropic tissue by reaction-diffusion equation [26]:

$$\frac{\partial V}{\partial t}=-(I_{ion}+I_{stim})/C_{m}+D(\frac{\partial^{2}V}{\partial x^{2}}+\frac{\partial^{2}V}{\partial y^{2}})$$

where V denotes the membrane potential, *I*_*ion*_ the sum of all ionic currents and *I*_*stim*_ is a stimulus current. D = 0.001 cm^2^/ms is the diffusion coefficient [27] and C_m_ = 1 μF/cm^2^ is the capacitance. We solved the model by using the operator splitter method [28] and the forward Euler method, where the time step was adaptively varying between 0.01 and 0.1 ms and the space step was 0.025 cm [29]. The tissue dimension was 15 cm × 15 cm. We initiated a spiral wave by applying the standard cross-field protocol: vertical field stimulation (S1) followed by the horizontal field stimulation (S2) in a coupling interval of 300 ms. Conduction velocity in this model is about 45 cm/s.

### Calculation of phase singularity

When spiral waves break and form multiple spiral waves, phase singularity (PS) can occur. Phase singularity (PS) is defined as a point which has an ambiguous phase, yet its neighboring sites show a continual phase progression from –π to +π [30]. Calculations of PS were performed by using the method of Iyer-Gray [31]. We provided the number of PS points in a unit time and space. For example, 0.4322 represents 0.4322 PS in a second and cm^2^, as our model results had spatial (225 cm^2^) and temporal (10 ms) resolution. The value 0.4322 was obtained by the following calculation: 1000 x 354 / 364 / 10 / 225 = 0.4322, where the area of the spatial domain is 15 cm x 15 cm = 225 cm^2^. If the PS was persistent and detected in all of the time frames, then there would be 364 PS in 364 time frames. This will correspond to a value of 1 (= 364/364) PS.

## Results

### Quantitative analyses for ion current contributions in APD

**Fig 1:**
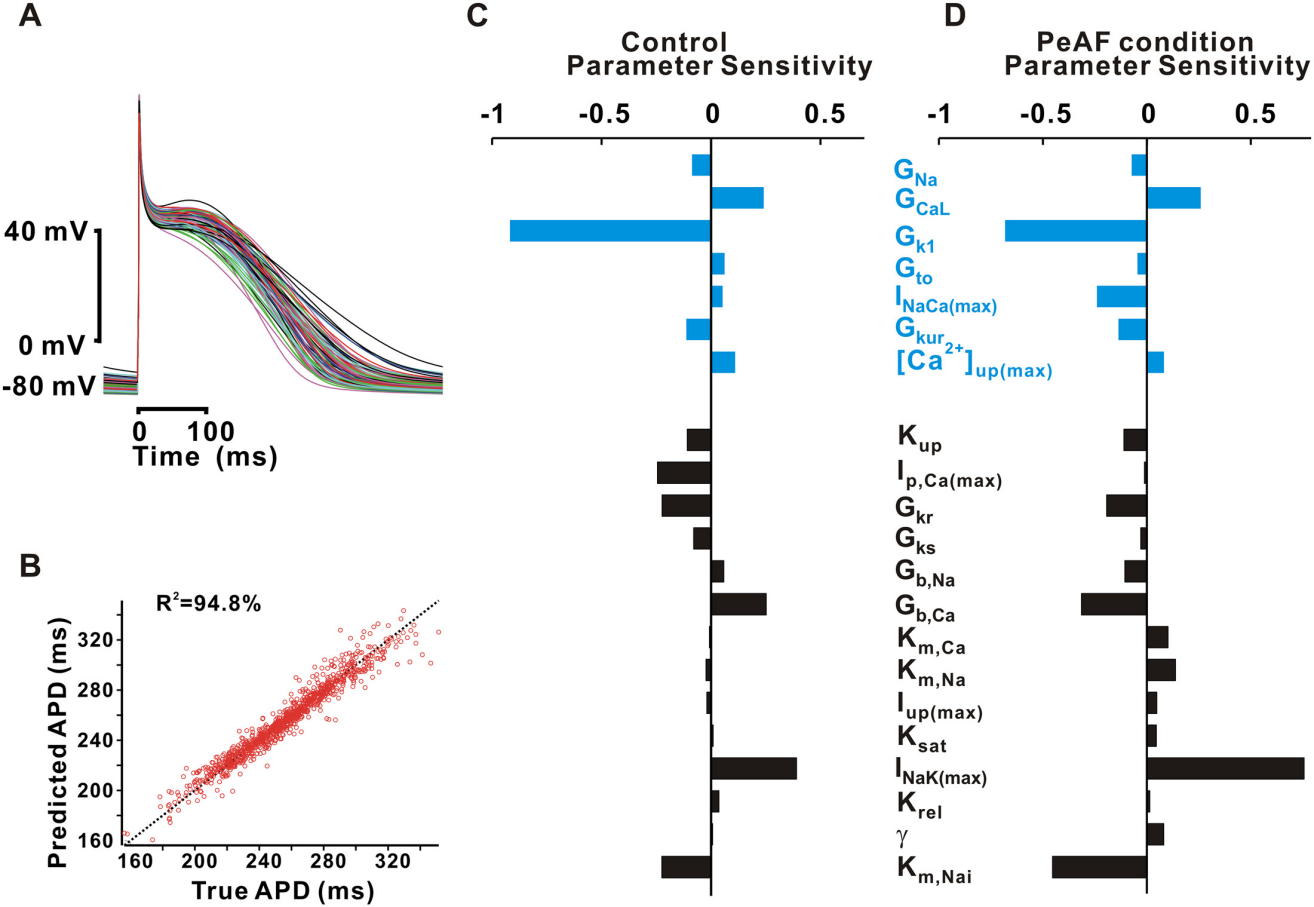
Parameter sensitivity analysis. (A) Simulation of AP by randomly varying model parameters (B) Prediction of APD by linear regression model with 94.8% correlation. Parameter sensitivities of model parameters on APD in control (C) and PeAF (D). Parameter group in the upper part were selected for ionic currents remodeled and the lower part not-remodeled in PeAF. Each value in the bar graph represents how change in a parameter influences on APD change. For example, G_K1_, the maximum conductance of I_K1_, showed the largest negative sensitivity in control. This implies that I_K1_ up-regulation will decrease APD.

To study how APD depends on changes in ionic currents, we calculated parameter sensitivities by regression analysis [20–22], with APD the primary model output. When the APD values obtained by numerical simulation were compared with the values predicted by regression analysis, the coefficient of determination (R^2^) between the two variables was 0.948 (Fig 1A and 1B). In control, sensitivity analysis showed that model parameters with the most influence on APD ranked in the order: G_K1_ > I_NaK(max)_ > G_CaL_ > G_Kr_ (Fig 1C). One surprising results from the analysis was the positive parameter sensitivity seen for I_NaK(max)_ because the Na^+^/K^+^ pump supplies outward current that acts to shorten rather than lengthen action potentials.

Since parameter sensitivities were calculated locally around the baseline parameters (Table 2), and some parameters in PeAF deviate substantially from the baseline value, we repeated the sensitivity analysis using the PeAF condition (Table 1) as the baseline model (Fig 1D). We displayed parameter sensitivities in two groups of parameters: PeAF-related remodeled (upper part) and not-remodeled (lower part). When we compared the ion current parameter sensitivity coefficients in the control and PeAF conditions, the majority of parameters showed similar directions and magnitudes in the bar graphs, indicating similar influences on the APD, but some exceptions were also found. Na^+^/Ca^2+^ exchange current (I_NaCa_), for instance, has a parameter sensitivity near zero in control, but a relatively large, negative parameter sensitivity in PeAF. This implies that blocking I_NaCa_ would have little effect in healthy atrial cells but produces AP prolongation in remodeled atrial cells. Another interesting difference is that in PeAF, I_NaK(max)_ is more influential than G_K1_ in determining APD, whereas the opposite is true in healthy atrial myocytes. In addition, K_m,Nai_, the intracellular Na^+^ affinity of Na^+^/K^+^ pump, showed the sixth biggest sensitivity in control, and the third biggest sensitivity in PeAF. This analysis therefore demonstrates that disease-induced remodeling can alter the relative importance of different ionic currents, and it shows how comprehensive parameter analysis can identify counterintuitive or surprising results.

### Enhancement of I_NaCa_ causes APD shortening during PeAF

**Fig 2:**
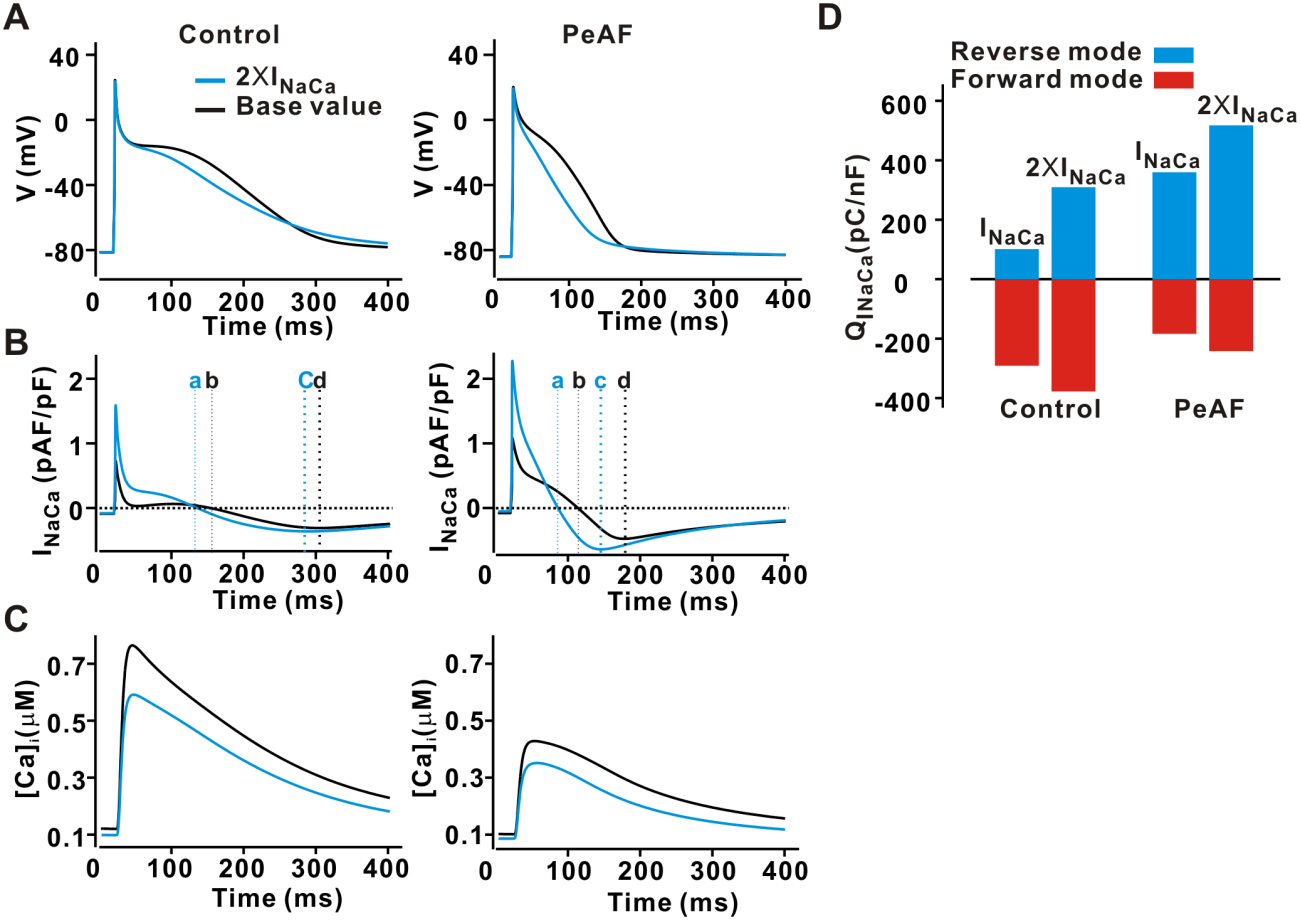
Effects of elevated I_NaCa_ on APD in control and PeAF. I_NaCa(max)_ was set to two times (+100%) (blue) the base value (black) for simulations. Superimposed action potentials (A), I_NaCa_ (B), and Ca^2+^ transients (C). Integrals of the forward and reverse mode I_NaCa_ in control and PeAF (D). We integrated the current I_NaCa_ from the time at which I_NaCa_ = 0 (denoted by **a** and **b** in 2B) to the time of AP repolarization, where I_NaCa_ has the minimum values (denoted by **c** and **d** in 2B).

To understand how a change in the maximum rate of I_NaCa_ during PeAF showed a negative parameter sensitivity (Fig 1), the model was paced to steady state at 1 Hz (250 beats) under four conditions: (1) control (Fig 2A, left, black); (2) control with 100% increase in I_NaCa(max)_ (Fig 2A, left, blue); (3) PeAF (Fig 2A, right, black); (4) PeAF with 100% increase in I_NaCa(max)_ (Fig 2A, right, blue). In control, an increase in I_NaCa(max)_ led to a minimal change in APD (Fig 2A, left). During PeAF, however, APD was substantially reduced with an increase in I_NaCa(max)_. Ionic current changes in PeAF led to reduced SR Ca^2+^ load and smaller Ca^2+^ transients (Fig 2C). Under these conditions, an increase in I_NaCa(max)_ led to a much larger change in reverse mode I_NaCa_, compared with the change in forward mode current. To quantify this, we separately integrated reverse mode and forward mode currents through Na^+^/Ca^2+^ exchange under the four conditions (Fig 2D). To calculate the net current in the forward mode, we integrated the current I_NaCa_ from the time at which I_NaCa_ = 0 (denoted by **a** and **b** in Fig 2B) to the time of AP repolarization, where I_NaCa_ has the minimum values (denoted by **c** and **d** in Fig 2B). Our results suggest that a relatively larger increase in reverse mode I_NaCa_ during PeAF could induce APD shortening when I_NaCa(max)_ is increased.

### Roles of I_K1_ and I_CaL_ in rate-dependent AP changes

**Fig 3:**
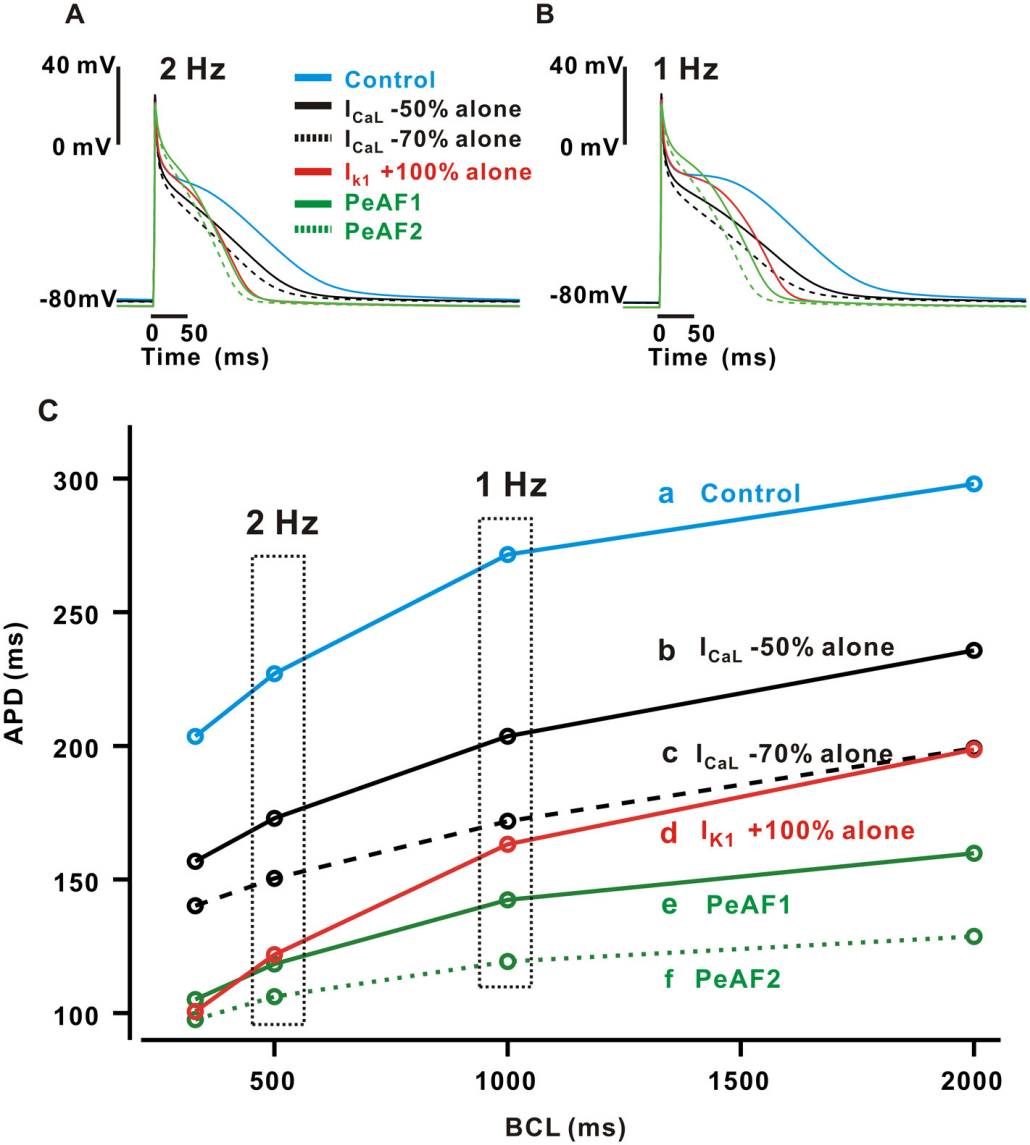
Simulation results of rate-dependent APD changes in six different conditions. APs were drawn in the steady-state condition at the pacing rates of 2 Hz (A) and 1 Hz (B). (C) The relationships between APD and BCL were drawn at the control condition (**a**), I_CaL_ decreased by 50% (**b**), 70% (**c**), I_K1_ increased by 100% (**d**), the PeAF condition with I_CaL_ decreased by 50% (**e**), and the PeAF condition (**f**) with I_CaL_ decreased by 70%.

We simulated the model under steady-state pacing, recording the APD after 250 s stimulation, at pacing rates from 0.5 Hz to 3 Hz (Fig 3). Based on previous studies suggesting 50% or 70% reduction of I_CaL_ and 100% increase of I_K1_ in PeAF [7, 16, 24, 32], we examined rate-dependent changes in APs under six conditions: (a) control, (b) 50% reduction in the density of I_CaL_ (I_CaL_ -50%), (c) 70% reduction in the density of I_CaL_ (I_CaL_ -70%), (d) 100% increase in the density of I_K1_ (I_K1_ +100%), and (e) the PeAF condition with I_CaL_ -50% (PeAF1), and the PeAF condition with I_CaL_ -70% (PeAF2). The last condition includes both I_CaL_ -50% and I_K1_ +100%, plus changes to five other channels that are remodeled in PeAF (Table 1). The action potentials shown in Fig 3A and 3B show that alterations caused by decreasing I_CaL_ are smaller than those caused by increasing I_K1_ and are similar at both fast (2 Hz) and slow (1 Hz) rates. Increasing I_K1_ by 100%, in contrast, is nearly able to approximate the PeAF condition at the fast rate (2 Hz), although at the slow rate additional ionic current changes clearly contribute to the observed AP alterations. Changing the resting potential affects the initial depolarization and final repolarization. For example, when the expression of I_K1_ is up-regulated, the resting membrane potential decreases, and this stabilizes the resting membrane potential by shortening the action potential duration [33]. In PeAF, in other words, the additional six ionic current alterations besides up-regulated I_K1_ seem to only contribute to altered AP shape during slow pacing. Interestingly, at 2 Hz, I_K1_ +100% alone almost accounts for the APD reduction of PeAF as mentioned in the above, but at 0.5 Hz both electrical remodeling in I_CaL_-70% alone and I_K1_+100% alone contribute equally to the APD reduction (Fig 3C). Note that the resting potential is in fact slightly more negative in PeAF cells than in control (-83.95mV vs. -81.40mV, see S1 Fig). The slope of APD vs BCL in PeAF between 0.5 Hz and 2 Hz becomes flatter (slope = 0.087 in control; 0.045 in PeAF) than that in control, reproducing the attenuated rate-dependent adaptation that has been observed experimentally [18]. Together these results suggest that the contributions of individual ionic currents to AP alterations depend greatly on pacing rate. In the next section, we assess the contribution of individual current to the rate-dependent APD change during the transition between two pacing rates.

### Role of I_CaL_ in the rate-dependent AP adaptation

**Fig 4:**
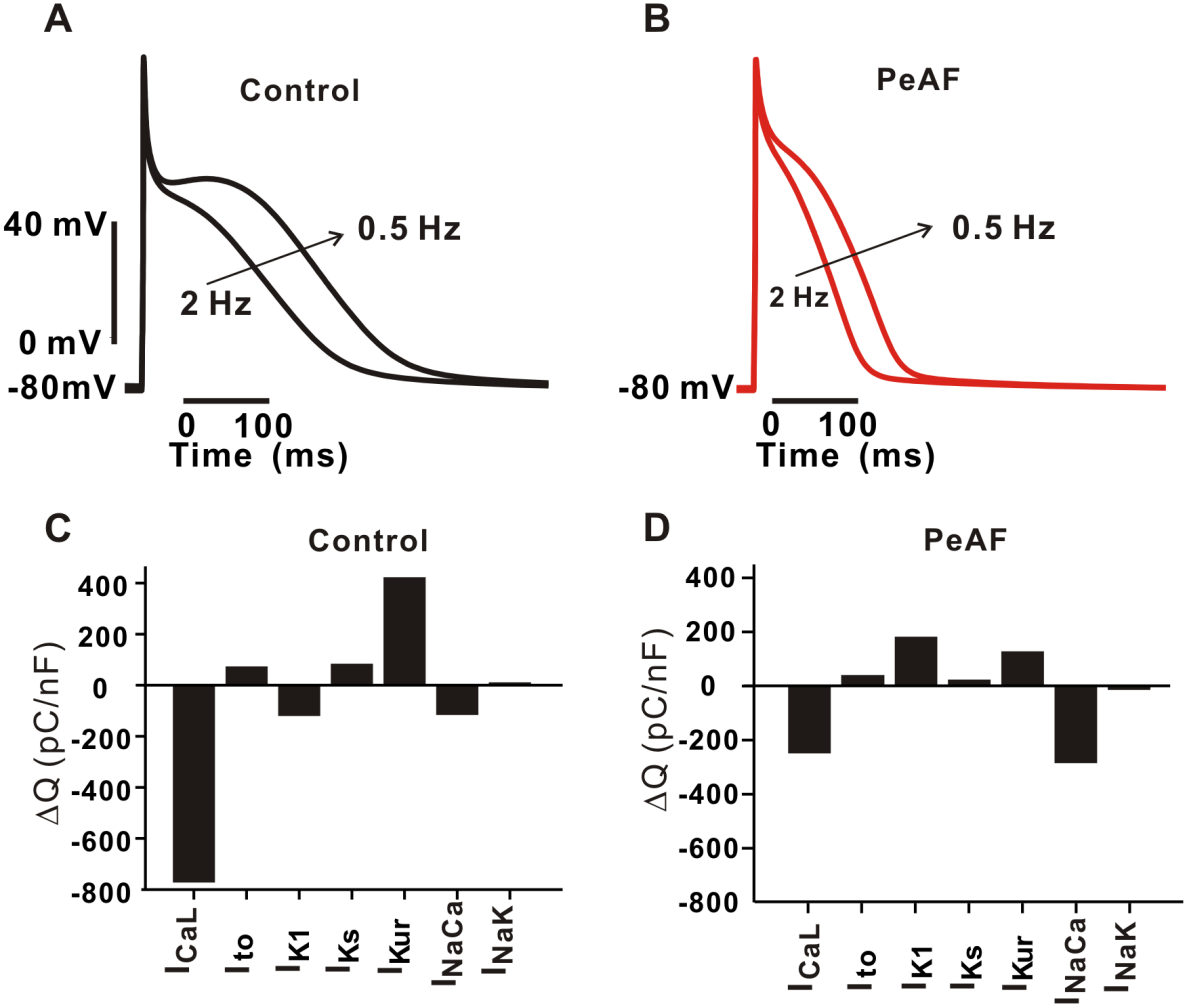
Quantifying the contribution of individual currents to rate-dependent APD adaptation. AP shapes changed during the transition between 0.5 Hz to 2 Hz in control (A) and the PeAF condition (PeAF1) (B). Evaluation of ionic current contribution during the transition from 2 Hz to 0.5 Hz in control (C) and PeAF (PeAF1) (D), where ΔQ is the total charge difference between 0.5 Hz and 2 Hz pacing rates. Negative value of ΔQ with I_CaL_ implies that more inward currents increased and contributed to the prolongation of APD from 2 Hz to 0.5 Hz. In other words, more depolarizing current decreased to lead to APD reduction during the transition from 0.5 Hz to 2 Hz.

Fig 3C shows the well-described phenomenon that faster pacing leads to shorter APs, which is further illustrated in Fig 4A and 4B, where we plot two APs seen during the transition from 2 Hz to 0.5 Hz under control and PeAF conditions. To better understand which ionic currents contribute to this rate-dependent AP change, and how this might be different in PeAF, we estimated the contribution of single currents to the AP by calculating the total charge difference (ΔQ) between the two rates. In control (Fig 4C), the negative, inward current I_CaL_ was much larger at 0.5 Hz than at 2 Hz (negative ΔQ), and it was therefore the dominant contributor to APD adaptation. In contrast, the outward current I_Kur_ was larger at 0.5 Hz than at 2 Hz, and rate-dependent changes in this current therefore opposed APD adaptation. During the PeAF condition (Fig 4D), the inward currents I_CaL_ and I_NaCa_ both contributed to the attenuated APD adaptation that was observed. Therefore, I_CaL_ plays the most important role in the rate-dependent APD adaptation in both control and PeAF conditions.

### Dynamics of reentry in 2D model depending on ion current remodeling of I_K1_ and I_CaL_

**Fig 5:**
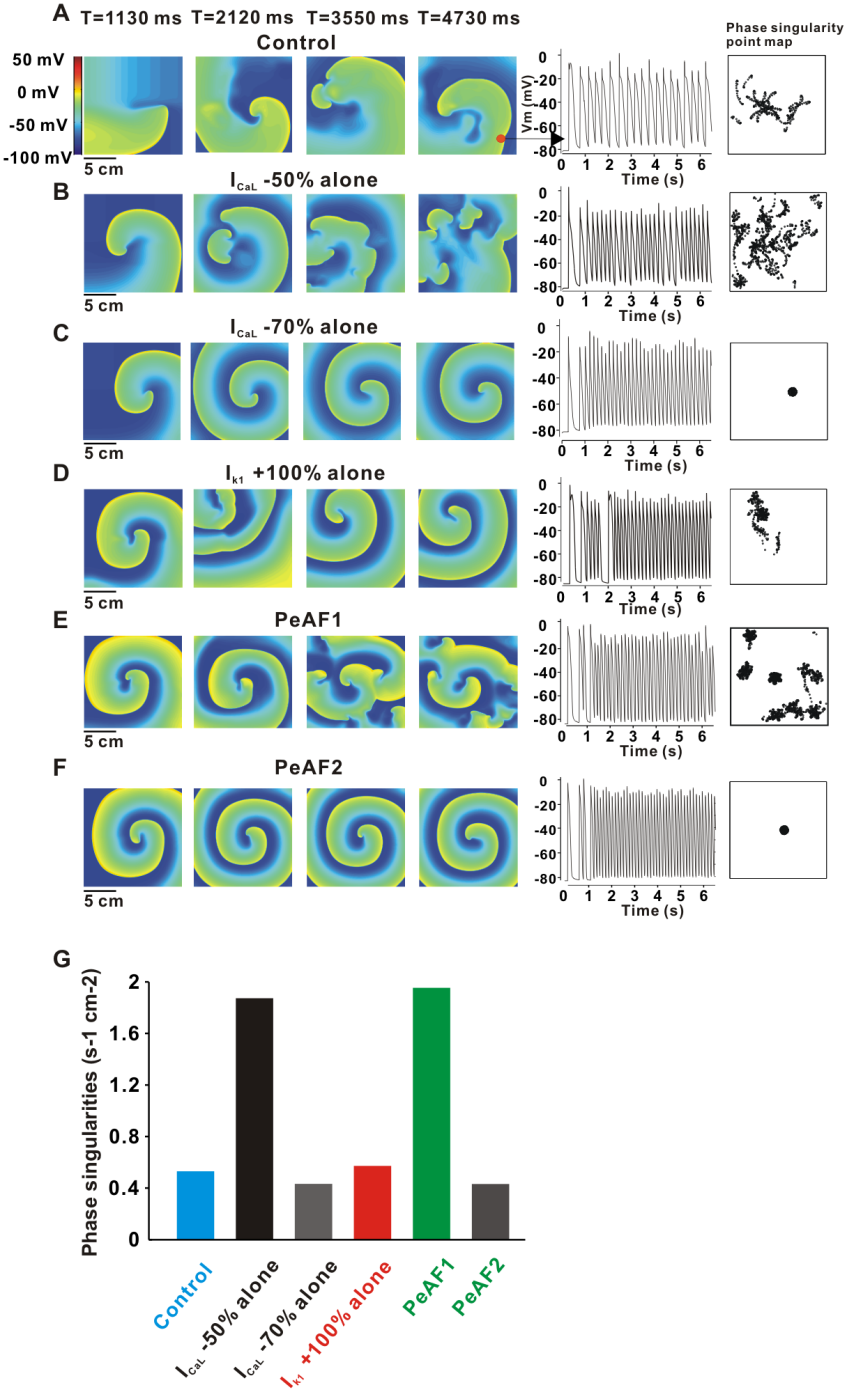
Emerging pattern formation of spiral waves in six different conditions. (A) control, (B) 50% reduction of I_CaL_, (C) 70% reduction of I_CaL_, (D) 100% increase of I_K1_, (E) PeAF condition with 50% reduction of I_CaL_ (PeAF1). (F) PeAF condition with 70% reduction of I_CaL_ (PeAF2). APs at the right and bottom corner (denoted by a red solid circle) were plotted to monitor the electrical activity of single cell. Phase singularity point maps (in the last column) were drawn to keep track of spiral cores. (G) Phase singularities in six different conditions. control (0.53 s^-1^cm^-2^), 50% reduction of I_CaL_ (1.87 s^-1^cm^-2^), 70% reduction of I_CaL_ (0.43 s^-1^cm^-2^), 100% increase of I_K1_ (0.57 s^-1^cm^-2^), PeAF1, PeAF condition with 50% reduction of I_CaL_ (1.95 s^-1^cm^-2^), PeAF2, PeAF condition with 70% reduction of I_CaL_ (0.4322 s^-1^cm^-2^).

To understand how electrophysiological differences in cells affect the spatial patterns observed when cells interact in tissue, we simulated spiral waves in 2D tissue under six conditions: control, I_CaL_ 50% down-regulation, I_CaL_ 70% down-regulation, I_K1_ 100% up-regulation, the PeAF1 condition (including both I_CaL_ 50% down-regulation and I_K1_ 100% up-regulation as in Table 1), and the PeAF2 condition (including both I_CaL_ 70% down-regulation and I_K1_ 100% up-regulation as in Table 1). The control case (Fig 5A) showed a quasi-stable spiral wave with transient wave breaks. For instance, the snapshot at 3550 ms in Fig 5A shows a moment soon after a wave break, when multiple wave-fronts are present. We plotted APs to describe electrical activities in the single cell located at the bottom-right corner of the domain (denoted by red solid circle). To describe the movement of the spiral cores, a phase singularity map was calculated and plotted in the last column.

In the case of I_CaL_ 50% down-regulation, a spiral wave broke up into small wavelets (Fig 5B). If I_CaL_ was decreased further, however (-70%), spiral waves were stabilized (Fig 5C). Up-regulation of I_K1_ by 100% also produced a sustained spiral wave with a higher frequency than control (Fig 5D). Interestingly, in the PeAF1 condition with 50% reduction of I_CaL_, the core of the spiral wave formed a persistent rotor at the center of the domain as well as wave breakups at the periphery forming small satellite rotors (Fig 5E). When a further reduction in I_CaL_ (-70%) was accompanied by other PeAF conditions as shown in Table 1 (denoted by PeAF2), a stabilized rotor was formed (Fig 5F). This pattern was similar to that in Fig 5C, but APs recorded at the bottom-right corner of the domain showed lower resting potentials than the case of Fig 5C, which might result from the remodeling in I_K1_ (+100%). This pattern resembled a mother rotor surrounded by daughter wavelets, as observed in other studies [34, 35]. Phase singularity maps showed a clear distinction among the five different conditions. In order to quantify what we observe in the figure, we counted the total number of phase singularities in the domain over 364 time frames from 1130 ms to 4760 ms. The average number of phase singularities were plotted as a bar graph in Fig 5G. There is one rotor in Fig 5C and 5F with different conditions, and this result suggests that the reduction in I_CaL_ (–70%) is a dominant factor for maintaining the stability of the spiral wave under the AF condition.

It has been suggested that there is a close link between APD restitution and spiral wave stability [36, 37], and we considered APD restitution in a 1D cable model, which is thought to be a good alternative model for studying the spatial and temporal aspects of spiral waves in a two-dimensional model [38]. We simulated the 1D cable model with 128 nodes (Δx = 0.025 cm), where 40 S1 stimulations were applied at one end for each BCL by decreasing BCLs from 600 ms until APD alternans or 2:1 block occurred. We measured APD_90_ (APDs) of the action potential at node 64.

**Fig 6:**
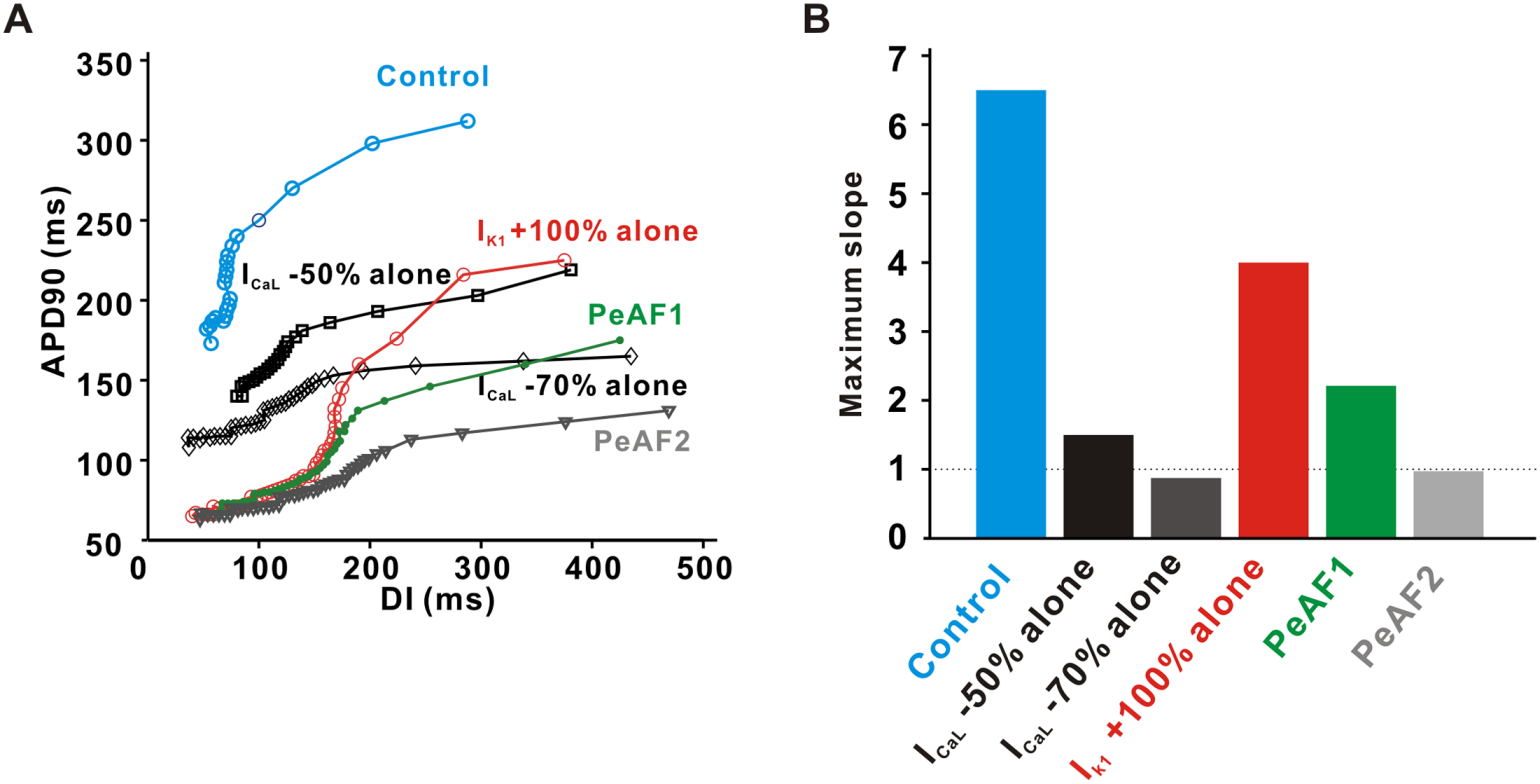
APD restitution curves and maximum slopes in a one-dimensional cable model. (A) The relation of the steady-state APDs (APD_90_) at varying basic cycle lengths (BCLs) were represented by the diastolic intervals (DI) for the one-dimensional cable model with 128 nodes (Δx = 0.025 cm). BCLs were progressively decreased by the steps of 600, 500, 400, 350, 320, 310, and 300 ms and further decreased by step of 5-ms until APD alternans or 2:1 block occurred. At each BCL, 40 stimuli were applied at one end in the cable model and used to calculate the steady-state APD in the middle of the cable. (B) The maximal slopes of APD restitution curves at six conditions are compared.

We compared the maximal slopes of the APD restitution curve in a one-dimensional cable model of the two cases, and found that the condition with I_CaL_ –50% alone had a larger slope (Smax = 1.5) than that with I_CaL_ –70% alone (Smax = 0.875; Fig 6). It has been suggested that the slope of APD restitution is an important determinant for spiral wave stability [36, 37]. This result suggests that a large reduction in I_CaL_ can stabilize spiral wave behavior by flattening the APD restitution curve. Among the six conditions, only two cases with I_CaL_ –50% alone and PeAF2 showed stable spiral waves without breakup. We measured the maximal slopes of APD restitution by fitting linear regression sequentially for every three points in DI [39]. This result showed that the slopes of APD restitution greater than 1 corresponded to the spiral wave dynamics with the first wave breakup (Fig 6).

## Discussion

In the current study, we systematically and quantitatively analyzed how parameters in an atrial cell model influenced AP dynamics in order to identify key ionic currents that contribute to electrical changes seen in PeAF, namely the shortening of APD and the attenuation of rate dependency. To achieve these goals, we performed several complementary analyses [20–22] on the mathematical model including: (1) sensitivity analysis to determine how model parameters affected APD; (2) calculations of transmembrane charge to assess how ionic currents influenced rate-dependent AP changes; and (3) analysis of 2D reentry dynamics to predict how changes seen in PeAF influenced the stability of rotors. Our quantitative analysis of an atrial cell model provides mechanistic insights into the rate-dependent APD change during AF.

### Mechanisms underlying effects of ionic current alterations in PeAF

Our sensitivity analysis provides a global view of how all model parameters influence physiology; a strength of this approach is that it identifies counterintuitive predictions that can be understood more completely through further mechanistic simulations. For instance, Fig 2 shows by the prediction that an increase in I_NaCa_ led to AP shortening only in the PeAF condition. Our mechanistic simulations showed that this resulted from an increase in the outward, reverse mode Na^+^/Ca^2+^ exchange (Fig 2). This non-symmetric increase in exchanger current occurred in part because Ca^2+^ transients were smaller in PeAF. Additionally, the ionic current alterations seen with the increase in I_NaCa_ in PeAF (Fig 2) depended in part on increase in intracellular [Na^+^]_i_ that occurred after either perturbation. In general, then, these results highlight the central role for changes in intracellular [Na^+^]_i_ in determining both APD and rate-dependent changes in AP shape, consistent with the results of prior studies [16, 40, 41].

### Mechanisms of rate-dependent APD shortening

The important cellular-level hallmarks of PeAF are AP shortening and attenuation of rate-dependent adaptation [3, 7, 42]. Some experimental studies have reported changes in ion channel expression, primarily either I_CaL_ down-regulation [7, 13, 43] or I_K1_ up-regulation [10, 11], as the underlying mechanisms of electrical remodeling in AF [16, 40]. Theoretical studies have also suggested that either I_K1_ [9, 12], I_CaL_ [16], or both [40] are important determinants of AP rate-dependency, and we confirmed these findings by a quantitative method called parameter sensitivity analysis. This approach has been applied in recent studies to understand atrial cell physiology and pathophysiology [40, 44]. Our analysis confirmed, as the previous studies suggested, that I_K1_ and I_CaL_ densities are the primary players in a reduction in APD. Two factors, which we can quantify separately, contribute to this: (1) the AP is relatively sensitive to changes in either parameter (Fig 1), and (2) the changes to these currents observed in PeAF are large compared with changes in other currents (e.g. 100% increase in I_K1_ compared with 10% decrease in I_Na_). At a particular pacing rate, individual current remodeling in I_K1_ or I_CaL_ led to large reductions in APD, which in some cases could nearly account for all the changes seen in PeAF (for instance the increase in I_K1_ at 2 Hz and decrease in I_CaL_ at 0.5 Hz).

Attenuation of rate-dependent adaptation in APD has been observed during steady-state pacing at cycle lengths > 400 ms [42, 45]. However, the degree of attenuation of APD rate-adaptation may vary greatly at various stages in AF remodeling and in different patient populations [5]. Our simulation result also reproduces the observation of a greatly reduced slope in the APD vs. BCL relation in the PeAF condition (Fig 3C). The underlying mechanisms of such rate-adaptation have not been fully understood, however. Remodeling of I_K1_ appears sufficient to approximate the APD reduction in PeAF at a fast rate (2 Hz), but this change alone is insufficient at slower rates (e.g. 1 Hz and 0.5 Hz). Thus, remodeling of additional currents contribute to changes in PeAF under these conditions. To identify which currents make the greatest contributions to APD adaptation during the transition between fast and slow pacing rates, we integrated individual currents and calculated the total charge difference between the 2 Hz and 0.5 Hz pacing rates. Interestingly, I_CaL_ and I_NaCa_ were primarily important in the determination of rate-dependent APD change. Although the role of I_CaL_ diminishes in PeAF because of the 50% reduction in its current density, I_CaL_ still plays a significant role in AP rate-dependency. In PeAF, I_NaCa_, which is up-regulated by 40%, also contributes to rate dependent AP changes, and this current is of course indirectly modulated by I_CaL_ through alterations in intracellular [Ca^2+^]. Thus, I_CaL_ is important in the determination of rate-dependent AP adaptation, although its role diminishes in PeAF, parallel to the attenuation of rate-dependent effects.

### I_K1_ and I_CaL_ alter reentry patterns during PeAF

As I_K1_ and I_CaL_ contributed significantly to APD shortening and rate-dependent AP adaptation at the single cell level, they also changed cell-to-cell interaction and spiral wave patterns in a 2D model. Compared to the control condition in a 2D tissue model, a 2-fold increase in I_K1_ seems to result in a sustained and stable spiral wave, as previously reported [14]. Interestingly, reduction in I_CaL_ could result in spiral wave breakup with multiple wavelets (Fig 5B), or a stable spiral wave (Fig 5C), depending on the degree of current down-regulation (50% reduction versus 70% reduction, respectively). Furthermore, a large reduction (-70%) of I_CaL_ in the condition of PeAF becomes a dominant factor that leads to a stable spiral wave in PeAF (Fig 5F). In the PeAF condition with I_CaL_ -50%, a persistent central mother rotor generated passive wave breakups and small daughter rotors at the periphery (Fig 5E). This modeling prediction is consistent with previous reports that both multiple wavelet type fibrillation [5] and mother rotor type fibrillation [46] can coexist in the same heart [47]. In summary, we found that a modest reduction in I_CaL_ induces spiral wave breakup, whereas a large reduction in I_CaL_ leads to stabilize spiral wave in PeAF.

### Limitations

To identify major ionic currents that are responsible for impaired APD shortening with increased pacing rates in PeAF, we used an atrial cell model and an isotropic monodomain 2D tissue model. We demonstrated by parameter sensitivity analysis that I_K1_, I_NaK_, I_CaL_ and I_Kr_ have the greatest effects on APD (Fig 1), but we limited our simulations of APD rate-adaptation to the changes in I_K1_ and I_CaL_ (Fig 3) because they show large parameter sensitivities on APD and are known to be altered largely in PeAF [32]. But this does not mean that we exclude a priori roles of other channels in APD rate adaptation. For instance, changes in repolarizing K^+^ currents (I_Kr_ and I_Ks_) have been proposed to become important at fast pacing rates [48, 49], and these effects may become accentuated by increases in extracellular [K^+^] [50], secondary to changes in extracellular [K^+^], have been proposed to become important at fast pacing rates. However, these phenomena were not explored in this study. Similarly, although atrial remodeling in AF combines both electrical and structural remodeling, we did not consider gap junction remodeling, interstitial fibrosis, collagen density change, or fiber orientation in this study. We showed that a relatively larger increase in reverse mode I_NaCa_ during PeAF could induce APD shortening with increasing in I_NaCa(max)_. This result may not be observed if a model has very little reverse mode of I_NaCa_ as Grandi et al. model does (See S2 Fig).

## Conclusions

Our systematic and quantitative analysis of a human atrial cell model confirms that increased I_K1_ amplitude during electrical remodeling in PeAF plays a dominant role in APD shortening, whereas I_CaL_ contributes significantly to rate-dependent AP adaptation in control and PeAF. This approach provides a counterintuitive but interesting prediction about the effect of up-regulated I_NaCa_ on APD in PeAF that can be further explored through the model simulation to obtain a mechanistic insight into the interactive roles of I_NaCa_ via the regulation of [Na^+^]_i_ in the APD shortening. Furthermore, simulation results with reentry patterns in tissue models suggest that a large reduction (-70%) in the amplitude of I_CaL_ can be a major factor for the formation of a stable spiral wave.

## Supporting Information

S1 Fig Resting membrane potentials (RMP) in control and PeAF. (TIF)

S2 Fig Comparisons between three models of I_NaCa_. (TIF)
