# Supplementary figures and images for: The Contribution of Ionic Currents to Rate-Dependent Action Potential Duration and Pattern of Reentry in a Mathematical Model of Human Atrial Fibrillation
Source: PLoS One. 2016 Mar 10;11(3):e0150779. doi: 10.1371/journal.pone.0150779 (PMC4795605; doi:10.1371/journal.pone.0150779)

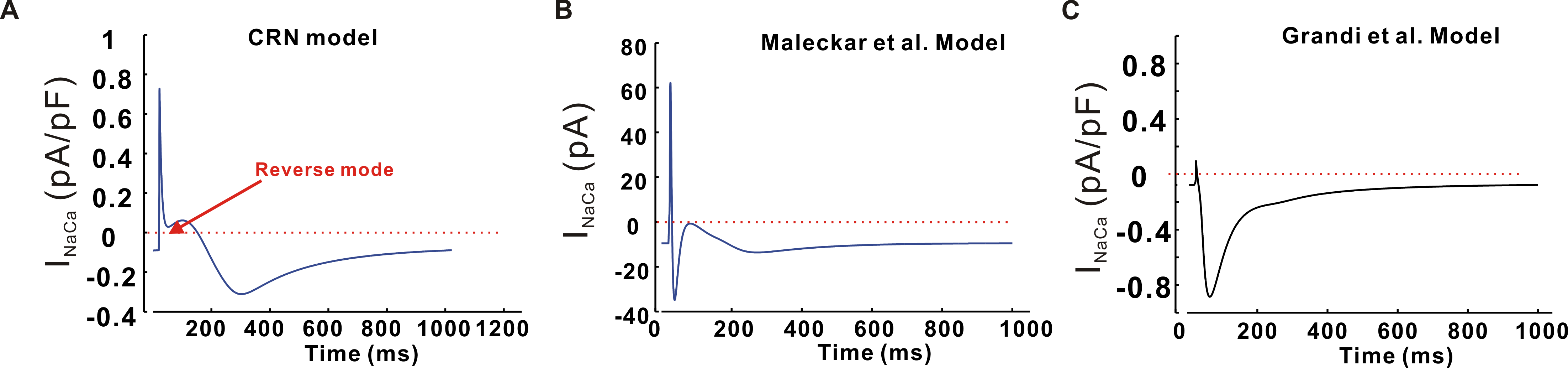

Supplement: S1 Fig — (TIF) [file pone.0150779.s001.tif]

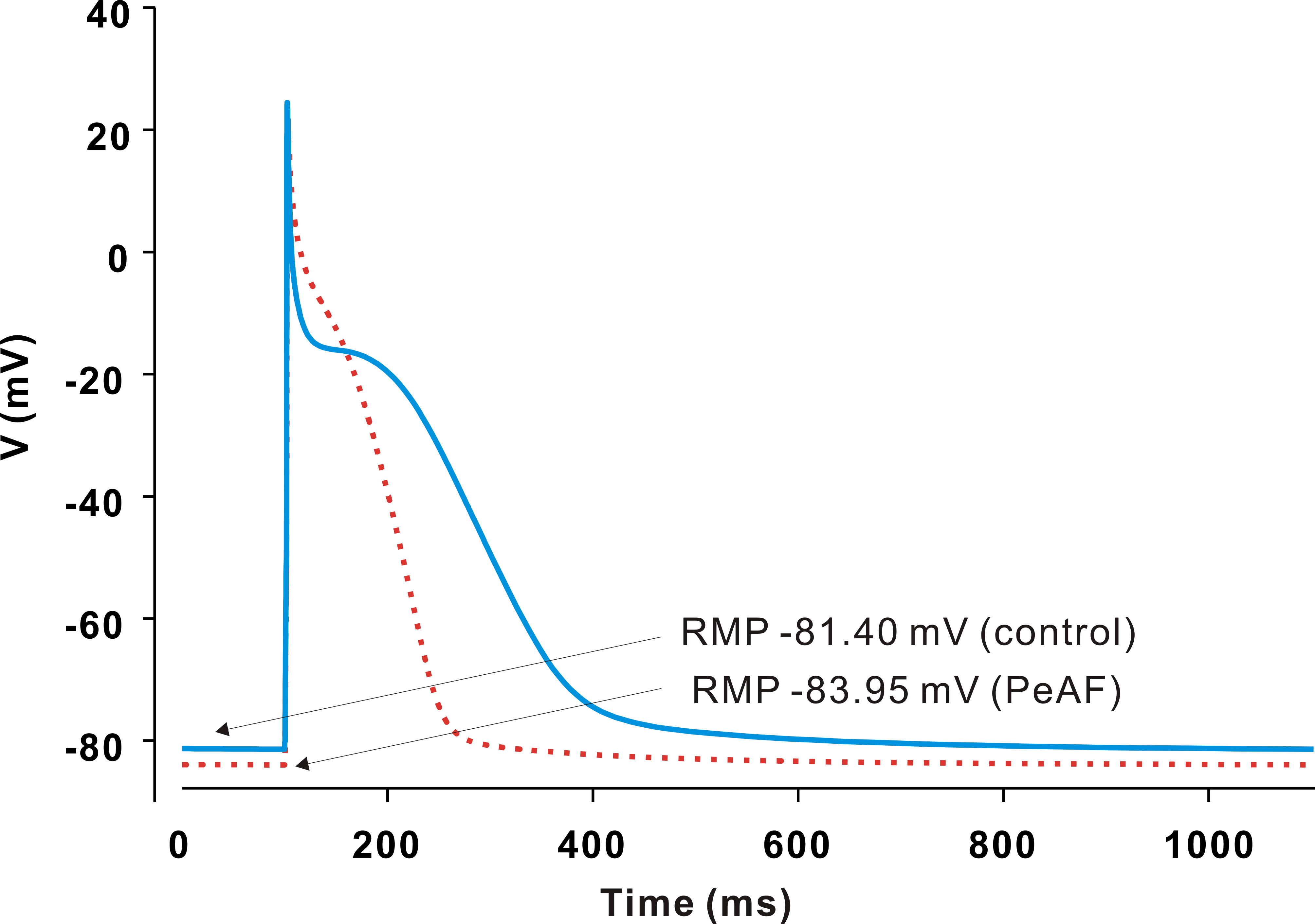

Supplement: S2 Fig — (TIF) [file pone.0150779.s002.tif]
